# Supplementary material for: High-resolution map of the Plasmodium falciparum genome reveals MORC/ApiAP2-mediated links between distant, functionally related genes
Source: Nat Microbiol. 2025 Jun 30;10(7):1665–83. doi: 10.1038/s41564-025-02038-z (PMC12221972; doi:10.1038/s41564-025-02038-z)
Supplement: Supplementary file 1 — Reporting Summary [file 41564_2025_2038_MOESM1_ESM.pdf]

nature portfolio

Corresponding author(s): Jessica Bryant

Last updated by author(s): 14/04/2025

# Reporting Summary

Nature Portfolio wishes to improve the reproducibility of the work that we publish. This form provides structure for consistency and transparency in reporting. For further information on Nature Portfolio policies, see our Editorial Policies and the Editorial Policy Checklist.

## Statistics

For all statistical analyses, confirm that the following items are present in the figure legend, table legend, main text, or Methods section.

| n/a | Confirmed | |
|---|---|---|
| ☐ | ☒ | The exact sample size (*n*) for each experimental group/condition, given as a discrete number and unit of measurement |
| ☐ | ☒ | A statement on whether measurements were taken from distinct samples or whether the same sample was measured repeatedly |
| ☐ | ☒ | The statistical test(s) used AND whether they are one- or two-sided<br>*Only common tests should be described solely by name; describe more complex techniques in the Methods section.* |
| ☒ | ☐ | A description of all covariates tested |
| ☒ | ☐ | A description of any assumptions or corrections, such as tests of normality and adjustment for multiple comparisons |
| ☐ | ☒ | A full description of the statistical parameters including central tendency (e.g. means) or other basic estimates (e.g. regression coefficient) AND variation (e.g. standard deviation) or associated estimates of uncertainty (e.g. confidence intervals) |
| ☒ | ☐ | For null hypothesis testing, the test statistic (e.g. *F*, *t*, *r*) with confidence intervals, effect sizes, degrees of freedom and *P* value noted<br>*Give P values as exact values whenever suitable.* |
| ☒ | ☐ | For Bayesian analysis, information on the choice of priors and Markov chain Monte Carlo settings |
| ☒ | ☐ | For hierarchical and complex designs, identification of the appropriate level for tests and full reporting of outcomes |
| ☐ | ☒ | Estimates of effect sizes (e.g. Cohen's *d*, Pearson's *r*), indicating how they were calculated |

*Our web collection on statistics for biologists contains articles on many of the points above.*

## Software and code

Policy information about availability of computer code

Data collection: BioRad Image Lab software 5.2

Data analysis:
cooler v0.9.3
hicstuff v3.2.4
hicrep 1.12.2 (R version)
pairtools v1.1.0
HiCExperiment 1.4.0
HiContacts 1.6.0
chromosight v1.6.3
bowtie2 v2.5.1
samtools v1.19.2
macs2 2.2.7.1
bedtools v2.31.0
ChIPseeker 1.40.0
deeptools 3.5.3
tidyCoverage 1.0.0
MEME 5.5.6
ggplot
DeepVenn (web interface)
STAR 2.7.11b
htseq-count 2.0.6

DESeq2 1.44.0
BioRad Image Lab software 5.2
FlowJo 10.9.0

All python packages were installed in python 3.10.12 with micromamba 1.5.1. All R packages were installed in R 4.4.0 with Bioconductor 3.19.

For manuscripts utilizing custom algorithms or software that are central to the research but not yet described in published literature, software must be made available to editors and reviewers. We strongly encourage code deposition in a community repository (e.g. GitHub). See the Nature Portfolio guidelines for submitting code & software for further information.

## Data

Policy information about availability of data

All manuscripts must include a data availability statement. This statement should provide the following information, where applicable:

- Accession codes, unique identifiers, or web links for publicly available datasets
- A description of any restrictions on data availability
- For clinical datasets or third party data, please ensure that the statement adheres to our policy

All data sets (ChIP-seq, RNA-seq, Micro-C) generated in this study are available in NCBI BioProject accession #PRJNA1146886.
Previously published data sets utilized in this study are available at the following NCBI accession numbers:
• AP2-I ChIP from (Santos et al., 2017)46: SRR5114665
• AP2-I ChIP Input from (Santos et al., 2017)46: SRR5114667
• TRZ ChIP from (Bertschi et al., 2017)43: SRR3085676
• TRZ ChIP Input from (Bertschi et al., 2017)43: SRR3085677
• HP1 ChIP from (Carrington et al., 2021)90: SRR12281320
• HP1 ChIP Input from (Carrington et al., 2021)90: SRR12281322
• ATAC-seq from (Toenhake et al., 2018)100: SRR6055333
• ATAC-seq from (Toenhake et al., 2018)100: SRR6055330
• ATAC-seq gDNA control from (Toenhake et al., 2018)100: SRR6055335
• Hi-C from (Ay et al., 2014)12: SRR957166
• Hi-C from (Subudhi et al., 2023)51: SRR19611536
• AP2-P ChIP from (Subudhi et al., 2023)51: SRR17171688
• AP2-P ChIP Input from (Subudhi et al., 2023)51: SRR17171686

## Research involving human participants, their data, or biological material

Policy information about studies with human participants or human data. See also policy information about sex, gender (identity/presentation), and sexual orientation and race, ethnicity and racism.

| | |
|---|---|
| Reporting on sex and gender | NA |
| Reporting on race, ethnicity, or other socially relevant groupings | NA |
| Population characteristics | NA |
| Recruitment | NA |
| Ethics oversight | NA |

Note that full information on the approval of the study protocol must also be provided in the manuscript.

# Field-specific reporting

Please select the one below that is the best fit for your research. If you are not sure, read the appropriate sections before making your selection.

☒ Life sciences ☐ Behavioural & social sciences ☐ Ecological, evolutionary & environmental sciences

For a reference copy of the document with all sections, see nature.com/documents/nr-reporting-summary-flat.pdf

# Life sciences study design

All studies must disclose on these points even when the disclosure is negative.

| | |
|---|---|
| Sample size | Three replicates were used for the early-stage Micro-C and four replicates for the late-stage Micro-C experiments. RNA-seq was performed in triplicate. Two biological replicates were performed for AP2-P and MORC ChIP-seq. Input material was used in quantities recommended by manufacturers or protocols. No sample size calculations were performed. Sample size was determined by what is recommended by the ENCODE standards. |

| | |
|---|---|
| Data exclusions | No data were excluded from the analysis. |
| Replication | Reproducibility of replicated Micro-C experiments was assessed by hicrep, by visual inspection and by computing Pearson correlation between replicates.<br>All western blots and DNA gels were performed/replicated at least twice independently, with the exception of ED Fig. 4H.<br>RNA-seq was replicated once.<br>ChIP-seq experiments were replicated twice independently (two biological replicates each).<br>For each Micro-C condition/stage, four technical replicates were performed. The late-stage Micro-C experiment was performed twice independently. |
| Randomization | Our study did not require randomization. Covariates are not relevant to this study. |
| Blinding | Our study did not require blinding. It did not involve animal or human cohorts or subjective counting. |

# Reporting for specific materials, systems and methods

We require information from authors about some types of materials, experimental systems and methods used in many studies. Here, indicate whether each material, system or method listed is relevant to your study. If you are not sure if a list item applies to your research, read the appropriate section before selecting a response.

## Materials & experimental systems

| n/a | Involved in the study | |
|---|---|---|
| ☐ | ☒ | Antibodies |
| ☐ | ☒ | Eukaryotic cell lines |
| ☒ | ☐ | Palaeontology and archaeology |
| ☒ | ☐ | Animals and other organisms |
| ☒ | ☐ | Clinical data |
| ☒ | ☐ | Dual use research of concern |
| ☒ | ☐ | Plants |

## Methods

| n/a | Involved in the study | |
|---|---|---|
| ☐ | ☒ | ChIP-seq |
| ☐ | ☒ | Flow cytometry |
| ☒ | ☐ | MRI-based neuroimaging |

## Antibodies

| | |
|---|---|
| Antibodies used | For western blot analysis, HA-tagged proteins, GFP-tagged proteins, and histone H3 were detected with anti-HA (Abcam ab9110, 1:1,000 in 1% milk-PBST) or anti-HA-HRP (Cell Signaling C29F4 HRP Conjugate #14031), anti-GFP (Chromotek PABG1) and anti-H3 (Abcam ab1791, 1:2,500 in 1% milk-PBST) primary antibodies, respectively, followed by donkey anti-rabbit secondary antibody conjugated to horseradish peroxidase ("HRP", Sigma GENA934, 1:5,000 in 1% milk-PBST). For ChIP-seq, HA-tagged proteins were immunoprecipitated with anti-HA (Abcam ab9110) antibody. |
| Validation | The anti-HA (Abcam ab9110, datasheet: https://www.abcam.com/en-us/products/primary-antibodies/ha-tag-antibody-chip-grade-ab9110?srsltid=AfmBOookpyx0G2pXgn6p2yEg5tCLpYY5Xa39t4DpGwrR0gUFLDoy1jdU#tab=datasheet) and anti-H3 (Abcam ab1791, datasheet: https://www.abcam.com/en-us/products/primary-antibodies/histone-h3-antibody-nuclear-marker-and-chip-grade-ab1791#tab=datasheet) are ChIP-grade antibodies that have been guaranteed by Abcam for ChIP and western blot. We have validated anti-HA and anti-H3 antibodies in previous studies (https://doi.org/10.15252/msb.20209569 and https://doi.org/10.15252/embr.202357090) for western blot analysis and ChIP-seq. The anti-GFP antibody (Chromotek PABG1, datasheet: https://www.ptglab.com/products/GFP-antibody-rabbit-polyclonal-PABG1.htm?srsltid=AfmBOoqVxarICqyRnVQMeS_sZpEvAZy112J4LetSdHbTfMXZXv0A5wuF#product-information) has been guaranteed by Chromotek for western blot analysis. We have validated the anti-GFP antibody for western blot analysis in a separate, unpublished story and would be happy to supply the data upon request. |

## Eukaryotic cell lines

Policy information about cell lines and Sex and Gender in Research

| | |
|---|---|
| Cell line source(s) | The wildtype cell line used for Micro-C is a clone of the NF54 reference strain. The parent cell line of all cell lines generated for this study is a bulk culture of 3D7 reference strain. |
| Authentication | We used PCR and Sanger sequencing to confirm correct integration of all epitope tag and glmS ribozyme sequences at our endogenous genes of interest. |
| Mycoplasma contamination | All strains have tested negative for mycoplasma. |
| Commonly misidentified lines<br>(See ICLAC register) | No commonly misidentified lines were used in this study. |

# Plants

Seed stocks: NA

Novel plant genotypes: NA

Authentication: NA

# ChIP-seq

## Data deposition

☒ Confirm that both raw and final processed data have been deposited in a public database such as GEO.

☒ Confirm that you have deposited or provided access to graph files (e.g. BED files) for the called peaks.

| | |
|---|---|
| Data access links<br>*May remain private before publication.* | All NextGen sequencing-based data sets (ChIP-seq, RNA-seq, Micro-C) generated in this study are available in NCBI BioProject accession #PRJNA1146886. |
| Files in database submission | We provide raw reads for ChIP-seq, RNA-seq and Micro-C experiments. We also provide processed files for Micro-C (.mcool), and ChIP-seq (.bigwig and .narrowPeak). |
| Genome browser session<br>(e.g. UCSC) | NA |

## Methodology

| | |
|---|---|
| Replicates | AP2-P and MORC ChIP-seq was performed in two biological replicates, with significant overlap between peaks identified by macs2. |
| Sequencing depth | AP2-P ChIP-Seq Replicate 1: 10191901 total reads, 10187115 unique mapped reads were recovered (paired-end, fragment size ~ 172); AP2-P ChIP-Seq Replicate 2: 6709230 total reads, 6701798 unique mapped reads were recovered (paired-end, fragment size ~ 152); MORC ChIP-seq Replicate 1: 6601103 total reads, 5624668 unique mapped reads were recovered (paired-end, fragment size ~ 196); MORC ChIP-seq Replicate 2: 38829706 total reads, 28029942 unique mapped reads were recovered (paired-end, fragment size ~ 218) |
| Antibodies | HA-tagged proteins (AP2-P and MORC) were immunoprecipitated with anti-HA (Abcam ab9110) ChIP grade antibody. |
| Peak calling parameters | Sequenced reads (150 bp paired end) were mapped to the P. falciparum genome (plasmoDB.org, version 3, release 56) using bowtie. PCR duplicates were filtered using samtools' fixmate and markdup commands and only alignments with a mapping quality ≥ 30 were retained (samtools view -q 30). The paired end deduplicated ChIP and input BAM files were used as treatment and control, respectively, for peak calling with the MACS2 subcommands. For each ChIP experiment, pileup files were first generated using the MACS2 pileup command, and the larger of the two files (i.e., Input or control) was down-sampled using MACS2 bdgopt. q-values and fold enrichment of ChIP/input was then calculated using MACS2 bdgcmp. Final peak calling was performed using MACS2 bdgcallpeak using a q-value cut-off of 0.001 (-c 3). For the two biological replicates of AP2-P and MORC, consensus peaks shared between biological replicates 1 and 2 were defined using the bedtools intersect command. |
| Data quality | All peaks used for analysis had a q-value < 0.001 and a fold enrichment > 2. 452 out of 946 AP2-P consensus peaks had a fold enrichment > 5. The 149 consensus MORC peaks had a fold enrichment > 5. |
| Software | bowtie2 v2.5.1<br>samtools v1.19.2<br>macs2 2.2.7.1<br>bedtools v2.31.0<br>ChIPseeker 1.40.0<br>deeptools 3.5.3<br>tidyCoverage 1.0.0<br>MEME 5.5.6<br>DeepVenn (web interface)<br><br>All python packages were installed in python 3.10.12 with micromamba 1.5.1. All R packages were installed in R 4.4.0 with Bioconductor 3.19. |

# Flow Cytometry

## Plots

Confirm that:

☒ The axis labels state the marker and fluorochrome used (e.g. CD4-FITC).

☒ The axis scales are clearly visible. Include numbers along axes only for bottom left plot of group (a 'group' is an analysis of identical markers).

☒ All plots are contour plots with outliers or pseudocolor plots.

☒ A numerical value for number of cells or percentage (with statistics) is provided.

## Methodology

| | |
|---|---|
| Sample preparation | Flow cytometry was used to determine parasitemia during a growth curve analysis. Two AP2-P-3HA-glms clones and a WT clone were tightly synchronized. Each culture was split, and glucosamine (Sigma G1514, 2.5 mM final concentration) was added to one half for approximately 96 h before starting the growth curve. The parasites were tightly re-synchronized and diluted to ~0.2% parasitemia (5% hematocrit) at ring stage. The growth curve was performed in a 96-well plate (200 µL culture per well) with three technical replicates per condition.. Every 24 h, 5 µL of the culture were fixed in 45 µL of 0.025% glutaraldehyde in PBS for 1 h at 4°C. After centrifuging at 800 g for 5 min, free aldehyde groups were quenched by re-suspending the iRBC pellet in 200 µL of 15 mM NH4Cl in PBS. A 1:10 dilution of the quenched iRBC suspension was incubated with Sybr Green I (Sigma S9430) to stain the parasite nuclei. |
| Instrument | CytoFLEX S cytometer (Beckman Coulter) |
| Software | FlowJo 10.9.0 |
| Cell population abundance | Red blood cells (RBC) range ~ 96%-99%<br>Singlets range ~ 96 %-99% of total RBCs identified population<br>Infected RBCs (FITC +) Range:  0.2%-4.5% of total singlets  population<br>Non-infected RBCs (FITC-) range: 95%-99.8% of total singlets population |
| Gating strategy | Red blood cells (RBCs)  were identified in a FSC and SSC comparison. Singlets were gated using FSC (H) vs. FSC (A) of previously identifed RBCs. P. falciparum infected RBC (iRBC) were selected inside the singlet (single) population based on FITC positive signal. |

☒ Tick this box to confirm that a figure exemplifying the gating strategy is provided in the Supplementary Information.
