# Supplementary figures and images for: High-resolution map of the Plasmodium falciparum genome reveals MORC/ApiAP2-mediated links between distant, functionally related genes
Source: Nat Microbiol. 2025 Jun 30;10(7):1665–83. doi: 10.1038/s41564-025-02038-z (PMC12221972; doi:10.1038/s41564-025-02038-z)

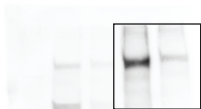

AP2-P-3HA

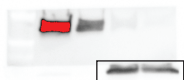

H3

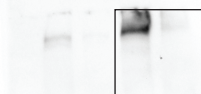

MORC-GFP

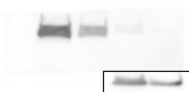

H3

Supplement: Supplementary file 18 — Unprocessed western blot for AP2-P. Unprocessed western blot for H3 loading control for AP2-P blot. Unprocessed western blot for H3 loading control for MORC blot. Unprocessed western blot for MORC. PDF showing where unprocessed western blots were cropped for Fig. 3a. [file 41564_2025_2038_MOESM18_ESM.pdf]

3B

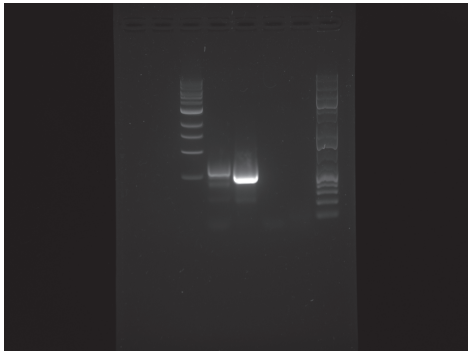

3C

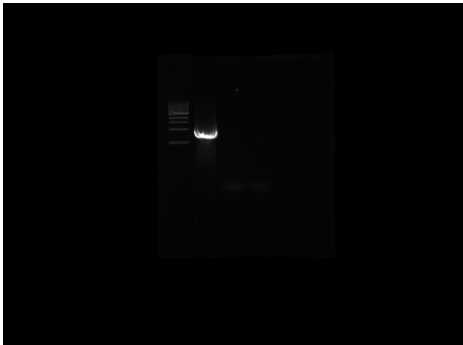

3D

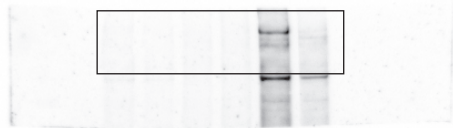

AP2-P-3HA

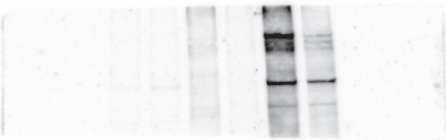

3E

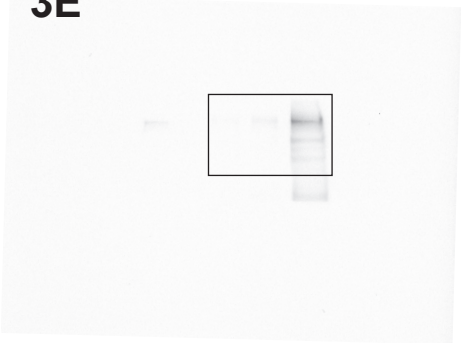

MORC-3HA

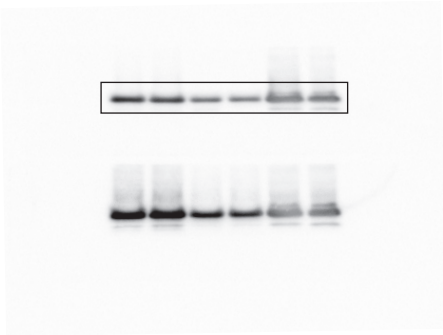

H3

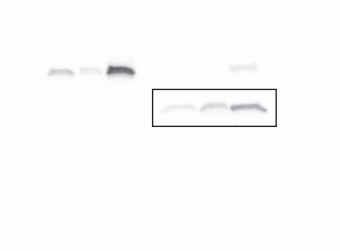

H3

Supplement: Supplementary file 19 — Unprocessed DNA gel. Unprocessed DNA gel. Unprocessed western blot for AP2-P. Unprocessed western blot for H3 loading control for AP2-P blot. PDF showing where unprocessed western blots were cropped for ED Fig. 3d. Unprocessed western blot for H3 loading control for MORC blot. Unprocessed western blot for MORC. PDF showing where unprocessed western blots were cropped for ED Fig. 3. [file 41564_2025_2038_MOESM19_ESM.pdf]

4A

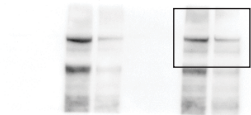

AP2-P-3HA

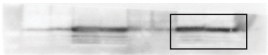

H3

4G

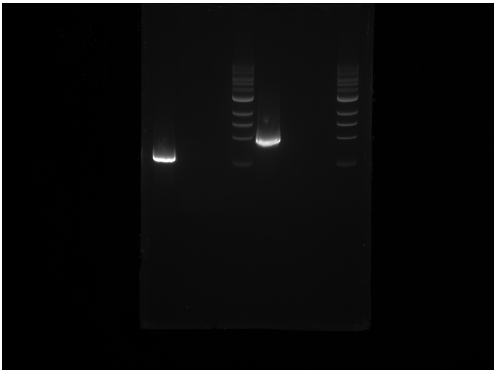

4H

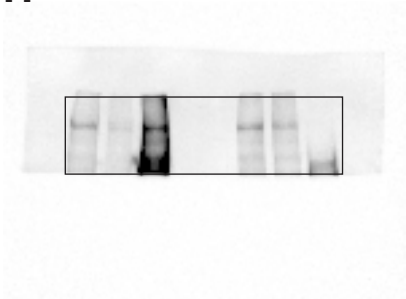

AP2-P-3HA

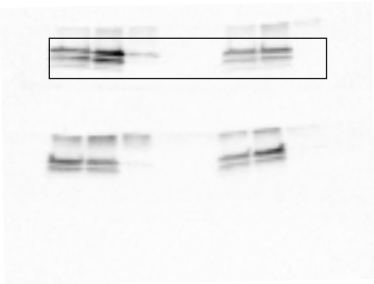

H3

4I

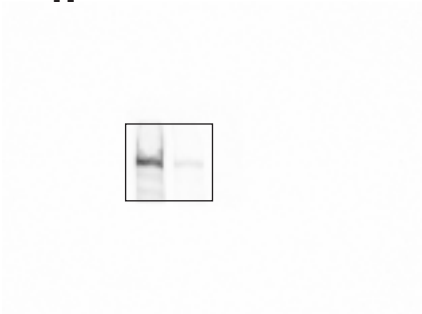

AP2-P-3HA

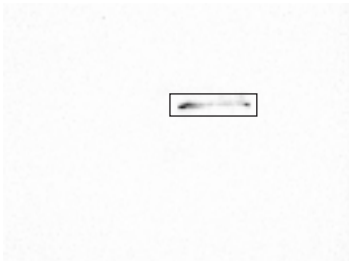

H3

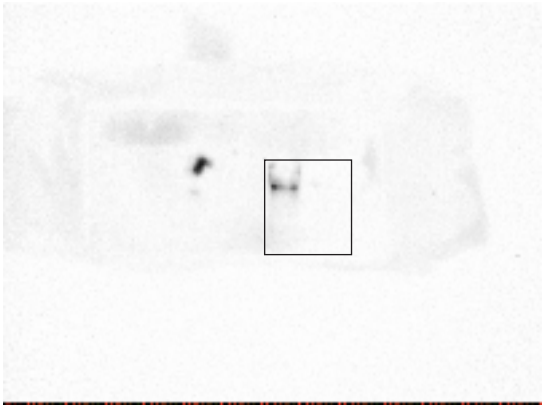

MORC-GFP

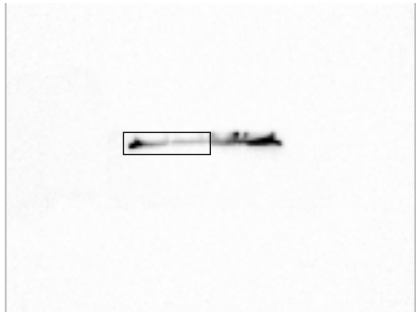

H3

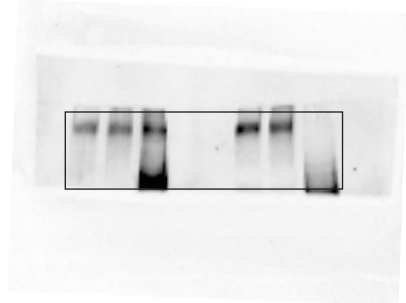

MORC-GFP

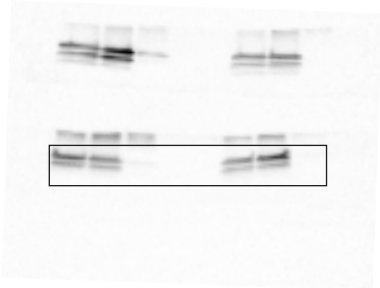

H3

Supplement: Supplementary file 20 — Unprocessed western blot for AP2-P. Unprocessed western blot for H3 loading control for AP2-P blot. PDF showing where unprocessed western blots were cropped for ED Fig. 4a. Unprocessed DNA gel. Unprocessed western blot for AP2-P. Unprocessed western blot for H3 loading controls for AP2-P and MORC blots. Unprocessed western blot for MORC. PDF showing where unprocessed western blots were cropped for ED Fig. 4h. Unprocessed western blot for AP2-P. Unprocessed western blot for H3 loading control for AP2-P blot. Unprocessed western blot for H3 loading control for MORC blot. Unprocessed western blot for MORC. PDF showing where unprocessed western blots were cropped for Extended Data Fig. 4i. [file 41564_2025_2038_MOESM20_ESM.pdf]
